# Correlation between perfluoroalkyl and polyfluoroalkyl substances and adult asthma in the NHANES

**DOI:** 10.1097/MD.0000000000050225

**Published:** 2026-08-28

**Authors:** Tiankai Shan, Kai Wu, Jingxian Jiang

**Affiliations:** c Department of Respiratory and Critical Care Medicine, The Fourth Affiliated Hospital of Soochow University (Suzhou Dushu Lake Hospital), Suzhou, Jiangsu, China; a Department of Cardiology, The First Affiliated Hospital of Soochow University, Medical University, Suzhou, Jiangsu, China; b Department of Pulmonary and Critical Care Medicine, Affiliated Hospital of Nantong University, Nantong, Jiangsu, China

**Keywords:** asthma, Bayesian kernel machine regression, negative association, PFAS mixtures, PFOS, weighted quantile sum regression

## Abstract

Per- and polyfluoroalkyl substances (PFAS) are pervasive organic compounds that have garnered significant attention due to their potential impacts on human health, particularly in relation to asthma, though current evidence remains limited. This study aimed to examine the association between PFAS exposure and adult asthma using a sample of 7106 participants aged 20 years and older from the NHANES survey, spanning 2003 to 2018. Survey-weighted multivariable logistic regression models, subgroup analysis, weighted quantile sum and Bayesian kernel machine regression models were employed to probe the relationship between PFAS and asthma. In the multivariable logistic regression analysis, higher log10-transformed perfluorooctane sulfonic acid (PFOS) and PFNA concentrations were significantly associated with lower odds of asthma (PFOS: OR = 0.76, 95% CI: 0.61–0.96; PFNA: OR = 0.77, 95% CI: 0.60–0.99). The inverse link between PFAS co-exposure and asthma incidence persisted across multi-pollutant models, weighted quantile sum regression, and Bayesian kernel machine regression models, with PFOS emerging as the primary negative influencer. In conclusion, this study highlights PFAS exposure is inversely associated with asthma risk in adults.

## 1. Introduction

Perfluoroalkyl and polyfluoroalkyl substances (PFAS), a class of synthetic chemicals known for their resistance to chemical degradation and high temperatures,^[1]^ have garnered attention. Among these, Perfluorooctane sulfonic acid (PFOS), and its derivatives, perfluorooctane sulfonyl fluoride, have been classified as persistent organic pollutants under the Stockholm Convention. Similarly, perfluorooctanoic acid (PFOA) and its precursors are also under consideration for this designation.^[2]^ The extensive production, application, and handling of PFAS over recent decades have led to global contamination and dispersion of these compounds, impacting aquatic and terrestrial ecosystems.^[3,4]^ Exposure to PFAS can occur through various routes, including ingestion of contaminated food and water, inhalation of indoor air, and dermal absorption.^[5]^ The prolonged elimination half-lives of most PFAS in the human body have intensified concerns regarding their widespread exposure and potential health risks.^[6]^

Asthma, a common chronic condition affecting individuals across all age groups, is characterized by airway inflammation and increased mucus secretion, leading to airway obstruction.^[7]^ Globally, asthma affects over 330 million individuals and imposes a substantial economic burden, particularly in the USA, where it impacts approximately 25 million people.^[8]^ Asthma accounts for about 495,000 deaths annually. Adult-onset asthma is more common in women, with low remission rates and relatively uncommon mortality. Adults with asthma often experience more frequent and unpredictable symptoms compared to those with childhood-onset asthma, including increased relapses and fewer periods of remission.^[9]^ While the etiology of asthma remains not fully elucidated, genetic predisposition and exposure to allergens and chemicals are recognized as pivotal factors in its pathogenesis and exacerbation.^[10,11]^ Numerous studies have confirmed a correlation between exposure to PFAS and outcomes related to asthma in children.^[12]^ Given the growing global environmental contamination, greater attention should be directed towards preventing adult-onset asthma, with an emphasis on the role of PFAS exposure.

In this study, we conducted a comprehensive analysis of data from the National Health and Nutrition Examination Survey (NHANES) spanning 2003–2018 to explore the relationship between PFAS exposure and adult asthma. To assess the associations between serum PFAS concentrations and asthma, we applied multivariable logistic regression models. Additionally, we employed weighted quantile sum (WQS) regression, Q-gcomp, and Bayesian kernel machine regression (BKMR) models to examine the overall associations between PFAS mixtures and asthma in adults. This research aimed to elucidate the associations between individual and combined PFAS exposures and the incidence of asthma among the adult population in the United States.

## 2. Materials and methods

### 2.1. Sample, research and design

**Figure 1.**
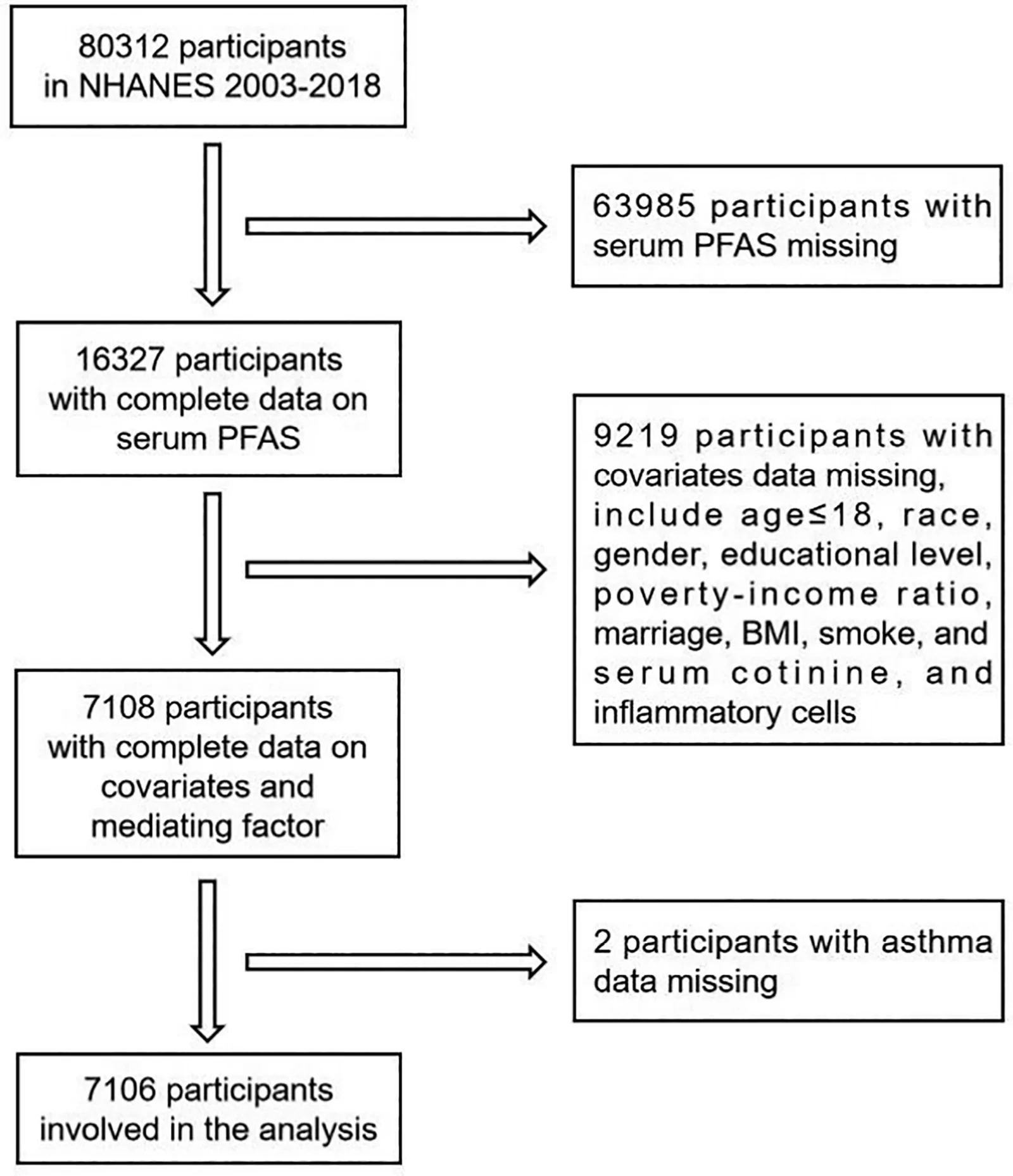
Chart depicting the process of selecting the study population. NHANES = National Health and Nutrition Examination Survey, PFAS = per- and polyfluoroalkyl substances, BMI = body mass index.

The NHANES sample population comprises noninstitutionalized civilian residents of the USA across various age groups. This study utilized publicly available data from 8 NHANES cycles spanning 2003 to 2004, 2005 to 2006, 2007 to 2008, 2009 to 2010, 2011 to 2012, 2013 to 2014, 2015 to 2016, and 2017 to 2018. After excluding participants with missing covariate data, a total of 7106 individuals with complete information on both asthma and PFAS exposure were included in the analysis. Additional methodological details are provided in Figure 1.

### 2.2. Measurement of phthalate metabolites

The study focused on serum PFAS exposure assessment due to higher concentrations and longer half-lives compared to urine, facilitating detection. This analysis primarily focused on 4 key compounds: PFOA, PFOS, PFNA and PFHxS. To quantify the total concentrations of PFOA and PFOS, the following calculations were employed: Total PFOA = linear perfluorooctanoate (n-PFOA) + linear perfluorooctanoate isomers (Sb-PFOA); Total PFOS = linear perfluorooctane sulfonate (n-PFOS) + Perfluoromethylheptane sulfonic acid isomers (Sm-PFOS). The limit of detection was determined with 95% confidence to surpass zero. To handle values below the limit of detection (LOD), NHANES analysis guidelines recommend using LOD; LOD/√2 serves as a proxy.

### 2.3. Definition of asthma

Data on asthma and its symptoms were systematically collected by trained interviewers using a standardized questionnaire. In accordance with the protocol established in a collective cohort study,^[13]^ individuals were classified as having a history of asthma if they answered affirmatively to the question, “Have you ever been diagnosed with asthma by a doctor or another healthcare provider?” Those who did not report a physician-diagnosed asthma served as the comparison group.

### 2.4. Covariates

Past research identified potential confounding factors linked to both PFAS exposure and asthma. These encompass factors such as participants’ age, race, gender, marital status, education, PIR, BMI, smoking habit, and serum cotinine levels. Careful consideration of these variables is essential to accurately assess the relationship between PFAS exposure and asthma in this study.

### 2.5. Mediation analysis

The study utilized mediation analysis to explore the role of eosinophils as mediators in the relationship between individual PFAS and asthma. Utilizing the eosinophil percentage, termed “Mediation,” we conducted causal mediation analysis to examine multiple components, including total effects, indirect effects, direct effects, and the proportion of mediation. In this analysis, each PFAS compound was treated as the exposure variable, eosinophil percentage served as the mediator, and asthma incidence was the outcome variable.

### 2.6. Statistical analysis

Because of the complex design of NHANES, appropriate weighting, clustering and stratification of the samples were implemented. The study adhered to NCHS recommendations, integrating the survey weights derived from the mobile exam center. Continuous variables following a normal distribution were represented as mean ± standard deviation and evaluated using the *t* test. For continuous variables that were not normally distributed, medians (with interquartile ranges: 25th and 75th percentiles) were reported, and comparisons were made using the Kruskal-Wallis H test. Categorical variables were denoted as percentages (%) or absolute values (n), and examination was conducted by the chi-square test. Given the skewed distribution of PFAS, a log10 transformation was applied to normalize the data before stratifying it into quartiles.

We performed both single-variate and multivariate logistic regression analyses to investigate the association between log10-transformed PFAS levels and asthma, with adjustments for potential confounders. No covariates were included in the crude model, while model I adjusted for age, race, gender, PIR, education, and marital status. Model II included adjustments for all covariates. Restricted cubic splines (RCSs) were employed to explore the dose-reaction relationship between log10-transformed PFAS and asthma. Subgroup analyses were also conducted to examine the consistency of the relationship across various population groups.

To effectively capture the combined impacts of co-occurring chemicals, the study employed both BKMR models and generalized WGS models implemented using the “bkmr” and “gWQS” packages, respectively. To ensure consistency across different multi-pollutant approaches, we also employed Q-gcomp as a complementary method to estimate the overall associations of PFAS mixtures with asthma. We iterated the mixture slope and model confidence bounds using 10,000 bootstraps. Q-gcomp was implemented using the R package “qgcomp.” The study employed a two-tailed significance threshold, with *P* < .05 considered statistically significant. R (version 4.2.0) was utilized to conduct statistical analyses. To account for survey weights and strata, the svyglm function from the “survey” package was utilized in the analysis.

## 3. Results

### 3.1. Baseline characteristics of participants

Table 1 summarizes data from the NHANES database spanning from 2003 to 2018, including a total of 7106 participants, with 1011 diagnosed asthma cases and 6095 individuals without asthma. Significant differences were observed between the 2 groups in terms of age, race, gender, PIR, BMI, and serum cotinine levels. However, no statistically significant differences were identified in marital status, educational level, or smoking behavior (*P* > .05).

**Table 1:** Baseline characteristics of study participants by incident asthma.

Characteristic	Non-Asthma, N = 6095*	Asthma, N = 1011*	*P* value
Age, n (%)			**.001**
≤40	2237 (39.7)	427 (45.6)	
40–60	1932 (36.2)	335 (36.6)	
>60	1926 (24.0)	249 (17.8)	
Gender, n (%)			**.005**
Male	3036 (50.0)	428 (43.4)	
Female	3059 (50.0)	583 (56.6)	
Race, n (%)			**.002**
Mexican America	1065 (8.6)	101 (4.8)	
Non-hispanic white	2689 (69.4)	510 (72.8)	
Non-hispanic black	1255 (10.7)	219 (10.8)	
Other Hispanic	474 (4.5)	88 (4.9)	
Other races	612 (6.7)	93 (6.7)	
Marriage, n (%)			.902
Married/living with a partner	3420 (56.6)	583 (57.4)	
Widowed/divorced/separated	1298 (20.7)	203 (20.0)	
Never married	1377 (22.7)	225 (22.7)	
Education, n (%)			.253
<High school	1529 (15.8)	211 (13.3)	
High school	1442 (38.3)	226 (36.9)	
>High school	3124 (45.9)	574 (49.8)	
Poverty income ratio, n (%)			**.031**
≤1.35	1974 (21.7)	396 (26.3)	
1.35–3	1867 (28.3)	275 (26.6)	
>3	2254 (49.9)	340 (47.1)	
BMI (kg/m2), n (%)			**<.001**
Normal	1860 (31.4)	239 (25.9)	
Obese	2148 (33.9)	485 (45.1)	
Overweight	2087 (34.7)	287 (29.0)	
Smoke, n (%)			.161
Current	1214 (20.7)	245 (23.7)	
Former	1426 (23.1)	252 (23.8)	
Never	3455 (56.2)	514 (52.5)	
Serum cotinine (mean (SD))	56.51 (125.78)	73.52 (140.30)	**.002**

* Continuous variables are presented as weighted mean ± standard deviation (SD), and categorical variables as weighted percentages (%). Participant counts are unweighted. Participant counts are unweighted. Percentages may not sum to 100% because of rounding.

### 3.2. Logistic regression to scrutinize the singular impact of PFAS exposure on asthma occurrence

The multivariable logistic regression analysis underscored the autonomous correlation between log10-transformed PFAS levels and asthma (Table S1, Supplemental Digital Content 1). A logistic regression analysis was performed to assess the association between individual PFAS compounds and asthma risk, chosen for its capacity to evaluate the direct impact of each PFAS while accounting for potential confounders. After adjusting for confounders in model 2, continuous log10-transformed PFOS and PFNA levels demonstrated an independent relationship with asthma risk (Table 2). Specifically, the OR for PFOS was 0.76 (95% CI: 0.61–0.96, *P* = .023), and for PFNA, the OR was 0.77 (95% CI: 0.60–0.99, *P* = .041).

**Table 2:** Association of PFOA, PFOS, PFHxS and PFNA with asthma using weighted multivariate logistic analysis.

	Crude model OR (95% CI)	Model 1 OR (95% CI)	Model 2 OR (95% CI)
PFOA			
Continuous	**0.74 (0.59, 0.93**)*	0.81 (0.62, 1.07)	0.87 (0.66, 1.14)
Quantile 1	Ref	Ref	Ref
Quantile 2	1.08 (0.84, 1.39)	1.15 (0.89, 1.51)	1.23 (0.93, 1.62)
Quantile 3	0.90 (0.72, 1.15)	0.99 (0.76, 1.28)	1.05 (0.80, 1.38)
Quantile 4	**0.76 (0.61, 0.95**)*	0.84 (0.64, 1.07)	0.89 (0.68, 1.15)
PFOS			
Continuous	**0.64 (0.53, 0.78**)***	**0.70 (0.56, 0.89**)**	**0.76 (0.61, 0.96**)*
Quantile 1	Ref	Ref	Ref
Quantile 2	**0.78 (0.61, 0.99**)*	0.83 (0.63, 1.07)	0.86 (0.66, 1.13)
Quantile 3	**0.63 (0.48, 0.81**)***	**0.68 (0.51, 0.90**)**	**0.73 (0.55, 0.96**)*
Quantile 4	**0.62 (0.48, 0.79**)***	**0.69 (0.52, 0.93**)*	0.75 (0.55, 1.00)
PFHxS			
Continuous	**0.70 (0.58, 0.87**)**	**0.79 (0.62, 1.00**)*	0.84 (0.66, 1.06)
Quantile 1	Ref	Ref	Ref
Quantile 2	0.90 (0.71, 1.15)	0.97 (0.76, 1.25)	1.00 (0.78, 1.30)
Quantile 3	**0.64 (0.50, 0.84**)**	**0.73 (0.53, 0.97**)*	0.76 (0.57, 1.01)
Quantile 4	**0.70 (0.54, 0.89**)**	0.76 (0.58, 1.01)	0.83 (0.63, 1.09)
PFNA			
Continuous	**0.65 (0.52, 0.83**)***	**0.73 (0.57, 0.93**)*	**0.77 (0.60, 0.99**)*
Quantile 1	Ref	Ref	Ref
Quantile 2	0.84 (0.66, 1.05)	0.87 (0.68, 1.11)	0.90 (0.71, 1.15)
Quantile 3	0.79 (0.63, 1.00)	0.84 (0.65, 1.09)	0.87 (0.67, 1.13)
Quantile 4	**0.62 (0.49, 0.79**)***	**0.68 (0.53, 0.87**)**	**0.73 (0.56, 0.93**)*

PFOA: Q1 (−1.15–0.18), Q2 (0.18–0.41), Q3 (0.41–0.62), Q4 (0.62–1.79); PFOS: Q1 (−0.85–0.68), Q2 (0.68–0.98), Q3 (0.98–1.25), Q4 (1.25–3.15); PFHxS: Q1 (−1.15–-0.10), Q2 (−0.10–0.18), Q3 (0.18–0.41), Q4 (0.41–1.68); PFNA: Q1 (−1.24–-0.30), Q2 (−0.30–-0.05), Q3 (−0.05–0.14), Q4 (0.14–1.91). Crude model: No covariates were adjusted. Model 1: Accounting for age, gender, ethnicity, marital status, educational level, and poverty income ratio. Model 2: Adjusted for all variables.

Bold values indicate statistically significant associations (*P* < .05).

95% CI = 95% confidence interval, OR = odds ratio.

* *P* < .05.

** *P* < .01.

*** *P* < .001.

Statistical significance was defined as a *P*-value <.05.

PFHxS = Perfluorohexane sulfonic acid, PFNA = Perfluorononanoic acid, PFOA = Perfluorooctanoic acid, PFOS = Perfluorooctane sulfonate.

PFAS levels were categorized into quartiles to generate categorical variables. In model 2, individuals in the Q3 category for PFOS had a reduced risk of asthma compared to those in the Q1 category (OR: 0.73, 95% CI: 0.55–0.96, *P* = .028), indicating a negative correlation between higher PFOS levels and asthma risk. Similarly, for PFNA, participants in the Q4 bracket exhibited a mitigated asthma risk compared to those in the Q1 category, with an OR of 0.73 (95% CI: 0.56–0.93, *P* = .013), reinforcing the inverse relationship between PFNA levels and asthma susceptibility.

RCS modeling is a widely utilized method in epidemiology and clinical trials for flexibly exploring nonlinear relationships within regression models. In this study, RCS was employed to investigate the potential nonlinear associations between PFAS exposure and asthma risk, which are critical for elucidating complex exposure-response patterns. As illustrated in Figure S1, Supplemental Digital Content 2, the RCS model revealed a linear relationship between log10-transformed PFOS and asthma likelihood after adjusting for all confounders (*P* for overall = .015, *P* for nonlinearity = .182). Furthermore, nonlinear relationships were analyzed for PFOA, PFNA, and PFHxS in relation to asthma risk, with all exhibiting an inverted *U*-shaped association with asthma. Overall, the results suggest that asthma risk is inversely correlated with PFOS levels (Figure S1, Supplemental Digital Content 2).

### 3.3. Subgroup and mediation analysis

A subgroup investigation was undertaken to validate the uniformity of the association between log10-transformed PFAS concentrations and asthma across various demographic categories. Subgroup analysis is critical for assessing the consistency of associations across different demographic groups, as it helps identify populations that may be more or less vulnerable to PFAS exposure. The segmentation encompassed age, race, gender, education, marital status, PIR, BMI, and smoking status. Interaction analyses revealed no significant interaction effects for any of these subgroups (all interaction *P* > .05), indicating that the association between PFAS and asthma was relatively consistent across all strata examined (Figure S2, Supplemental Digital Content 3). In summary, the relationship between PFAS exposure and asthma appears robust and not substantially modified by these factors.

Mediation analysis was performed to investigate whether the relationship between PFAS exposure and asthma is mediated by eosinophils, potentially providing insights into the biological pathways involved. As illustrated in Table S2, Supplemental Digital Content 4, the analysis revealed no significant mediation by eosinophils in the association between PFAS and asthma. For PFOA: the proportion mediated was −0.0694, the average causal mediation effects (ACME) were observed as follows: for PFAS1, the ACME was −0.001 (95% CI: −0.004 to 0.001, *P* = .209); for PFOS, the point estimate was 0.072, resulting in an ACME of −0.002 (95% CI: 0.004 to 0.000, *P* = .084); for PFHxS, the point estimate was 0.1593 with an ACME of −0.003 (95% CI: −0.005 to −0.001, *P* = .008); and for PFNA, the point estimate was 0.044 leading to an ACME of −0.001 (95% CI: −0.003 to 0.001, *P* = .491).

### 3.4. WQS model to analyze the relationship between PFAS co-exposure and the incidence of asthma

The WQS regression model was used to assess the overall correlation between multiple PFAS exposures and asthma, offering insight into how co-exposure to various PFAS compounds impacts health outcomes. In comparison to non-asthmatic adults, the log10-transformed concentrations of all 4 PFAS compounds were significantly lower in individuals with asthma (Figure S3, Supplemental Digital Content 5), and these compounds exhibited significant correlations with each other (all *P* < .001) (Figure S4, Supplemental Digital Content 6). The strongest correlations were identified between log10-transformed PFOA and log10-transformed PFOS (*R* = 0.730) and between log10-transformed PFOA and log10-transformed PFNA (*R* = 0.701).

**Figure 2.**
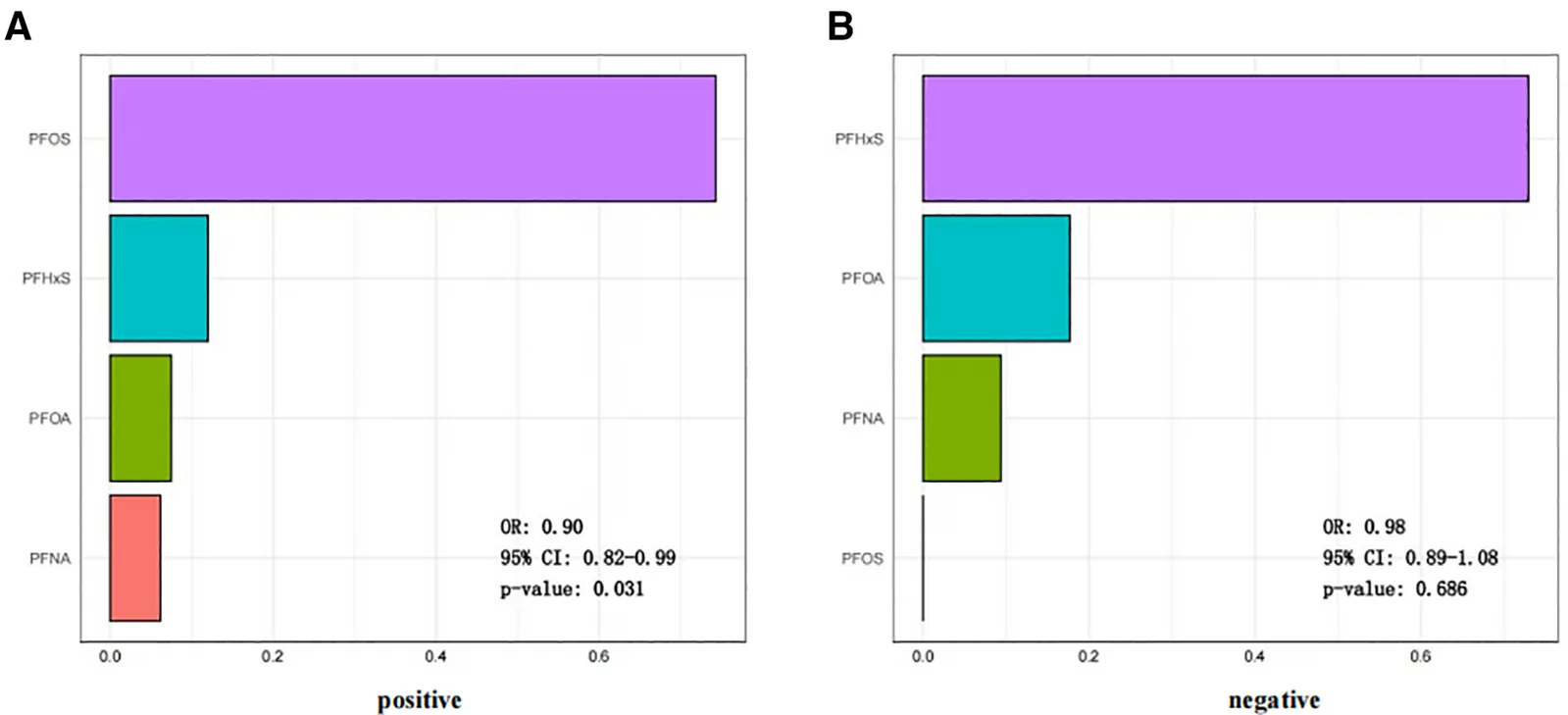
β (95% CI) of asthma associated with co-exposure to PFAS mixtures by WQS analyses. Models were adjusted for all covariates. CI = confidence interval, OR = odds ratio, PFAS = per- and polyfluoroalkyl substances, WQS = weighted quantile sum.

In the fully adjusted WQS regression model, a negative correlation was observed between the WQS index and asthma (OR [95%CI]: 0.90 [0.82, 0.99], *P* = .031) (Fig. 2). The estimated weights of the 4 PFAS compounds are presented in Table S3, Supplemental Digital Content 7, with PFOS being the most heavily weighted metabolite (weighted 0.743). However, no significant association was observed between the WQS index and asthma when assuming all beta coefficients were negative (Fig. 2).

### 3.5. BKMR and Quantile-based g-computation model to analyze the relationship between PFAS co-exposure and asthma incidence

**Figure 3.**
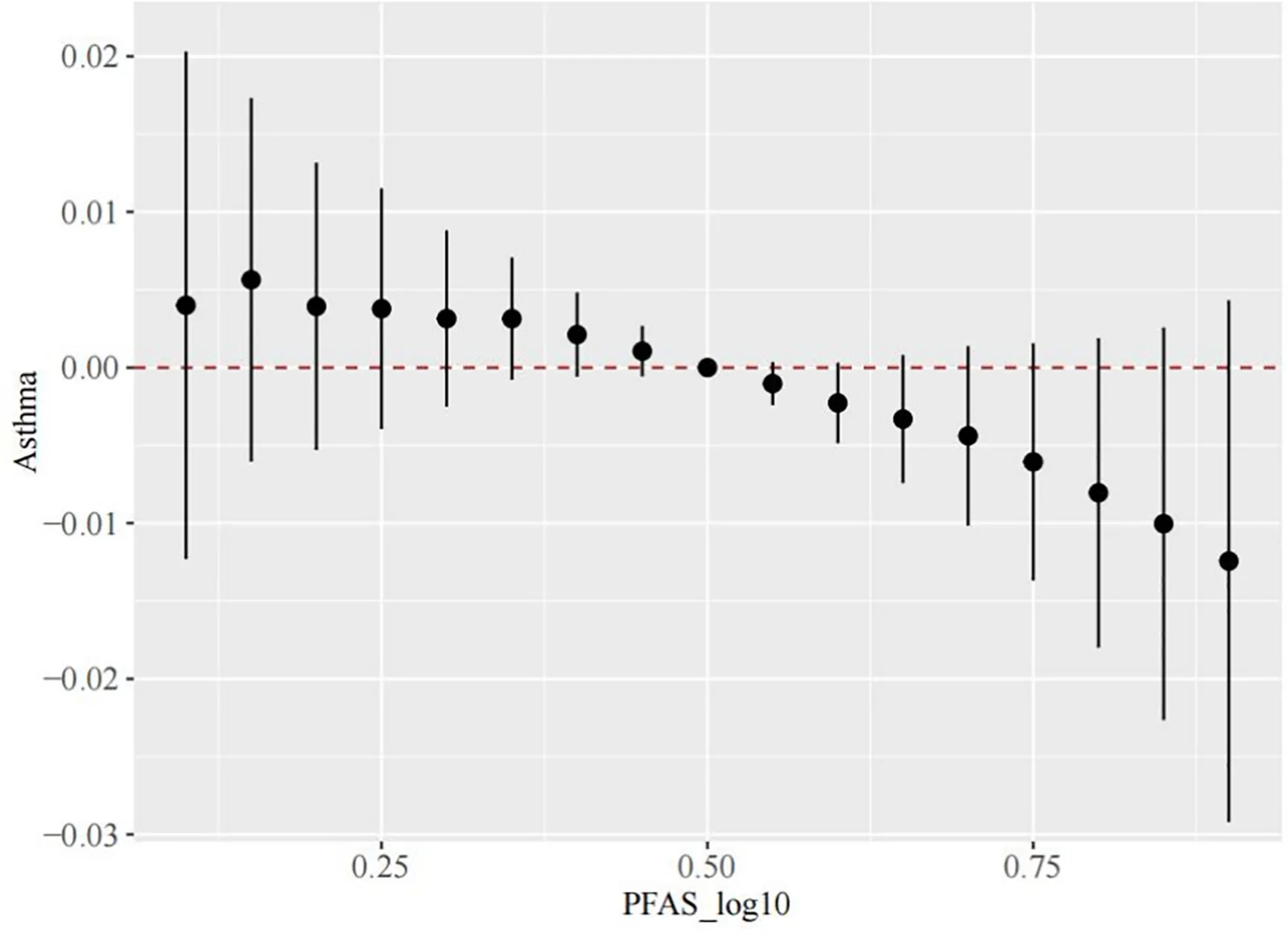
Overall connections between the combination of 4 PFAS and asthma among adults in Bayesian kernel machine regression models. This plot illustrates the estimated disparity in asthma occurrence. When PFAS combinations were situated at specific percentiles (between the 25th and 75th percentiles) as opposed to being maintained at the 50th percentile. We adjusted the model for all covariates. PFAS = per- and polyfluoroalkyl substances.

**Figure 4.**
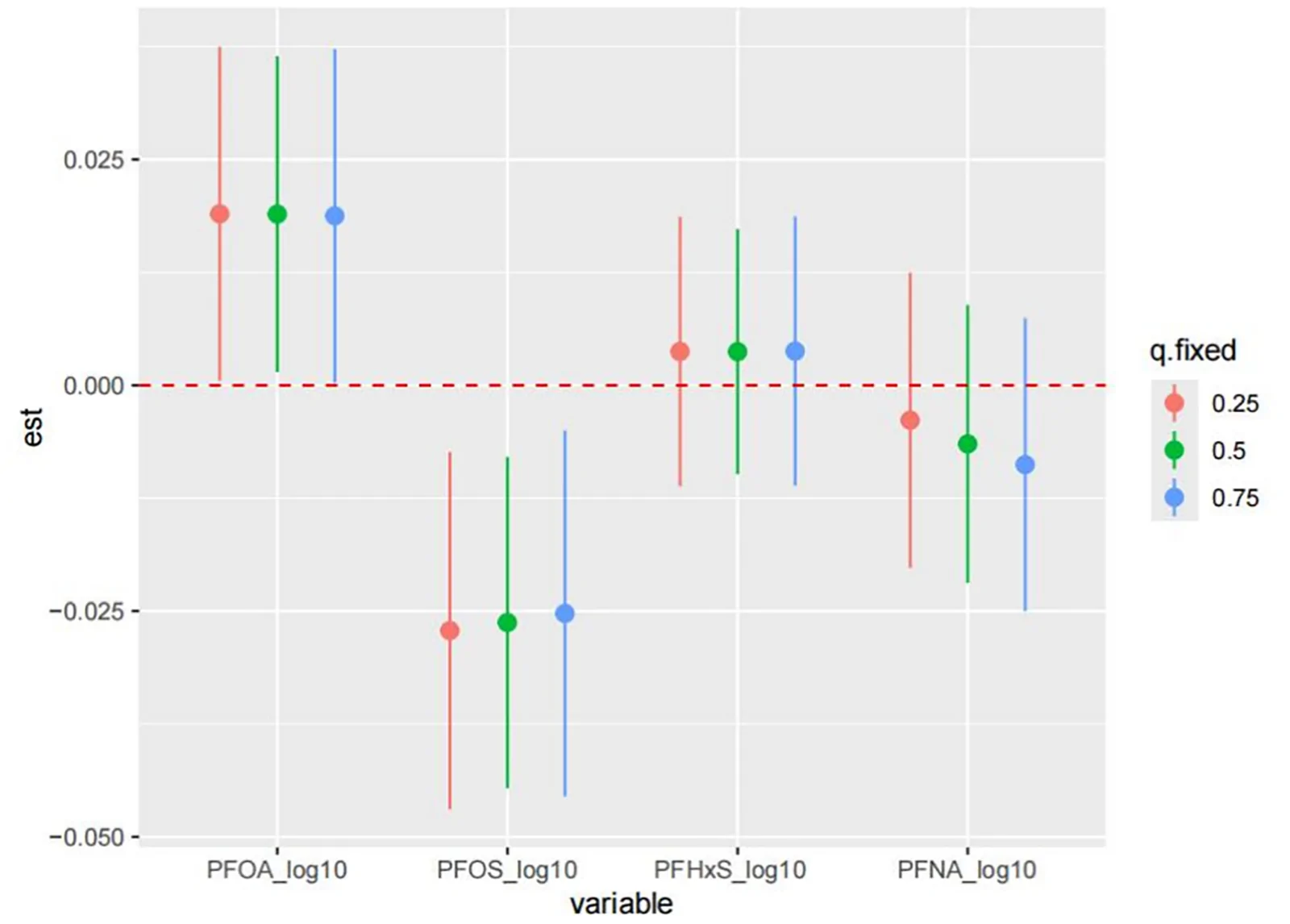
Links of each individual PFAS with sasthma among aldults in Bayesian kernel machine regression models. This graph illustrates the estimated variation in asthma associated with changes in individual PFAS from the 25th to 75th percentile, while holding all other PFAS constant at either the 25th (red line), 50th (green line), or 75th percentile (blue line). Dots represent the estimate, while horizontal lines show the 95% CrI. We adjusted the model for all covariates. PFAS = per- and polyfluoroalkyl substances.

The BKMR model offers a robust and flexible approach for estimating multivariable exposure-response functions, allowing for the selection of variables from potentially high-dimensional exposure vectors. This model is particularly effective in identifying interactions and nonlinear relationships among mixture components, especially within the context of highly correlated exposures. In this study, we employed BKMR to explore the complex, non-linear interactions among multiple PFAS compounds in relation to asthma risk, thereby providing a deeper insight into the effects of co-exposure. As illustrated in Figure 3, the overall impact of 4 PFAS mixtures on asthma risk was examined. While no statistically significant differences were observed at high PFAS concentrations compared to the 50th percentile, a discernible downward trend in asthma risk was noted across the general population (Fig. 3). Among the PFAS compounds, PFOS demonstrated the highest posterior inclusion probability (PIP = 0.6288) (Table S4, Supplemental Digital Content 8). Notably, PFOA showed a significant positive association with asthma risk when PFAS concentrations were fixed at the 25th, 50th, and 75th percentiles, whereas PFOS exhibited a negative correlation with asthma under the same conditions (Fig. 4).

**Figure 5.**
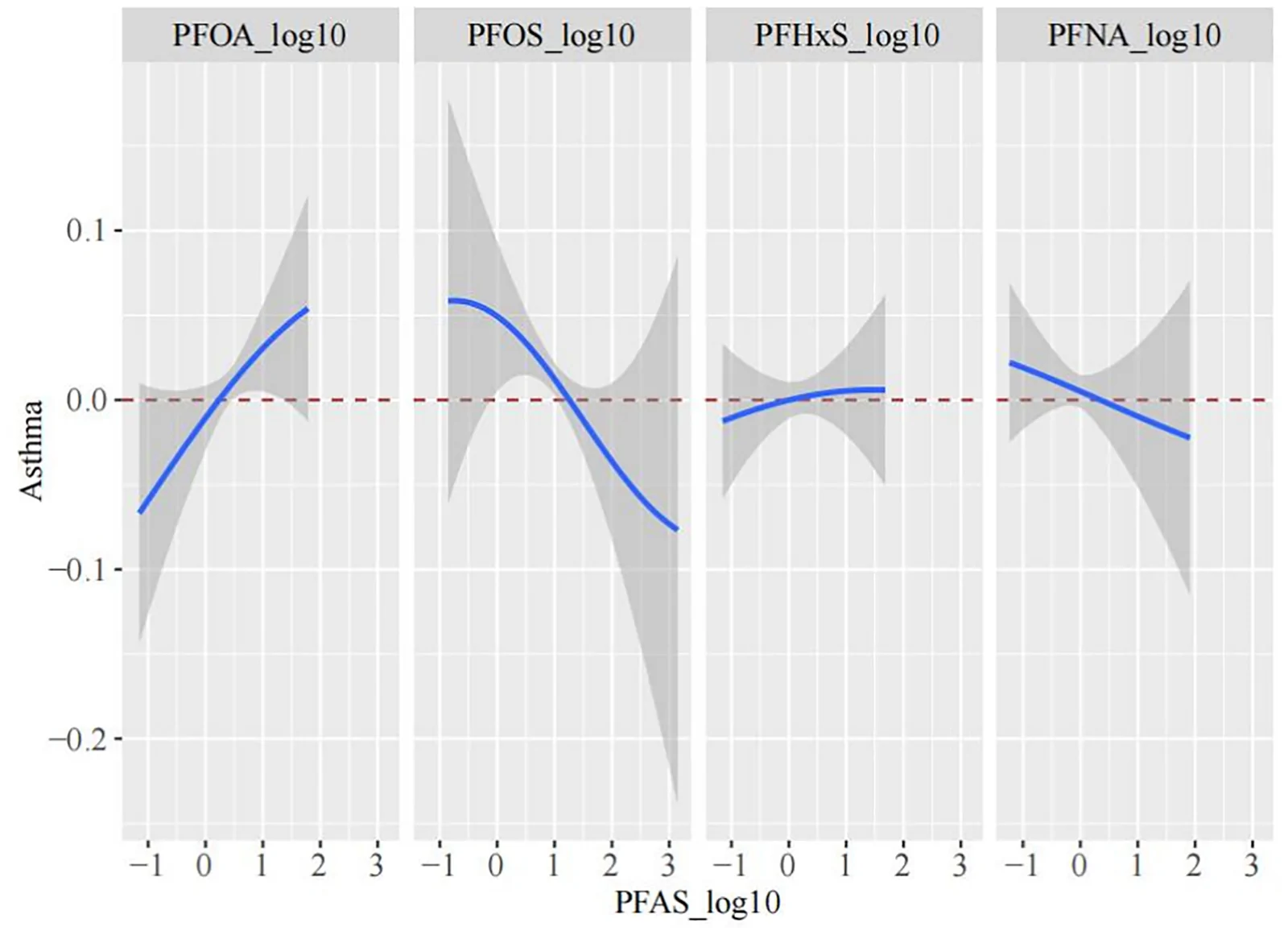
The univariate exposure-response functions and 95% credible intervals (shaded regions) between the levels of each PFAS and risk of asthma while fixing the levels of residual PFAS at the median, resulting from the BKMR model. The model was adjusted for all covariates. BKMR = Bayesian kernel machine regression, PFAS = Per- and polyfluoroalkyl substances.

**Figure 6.**
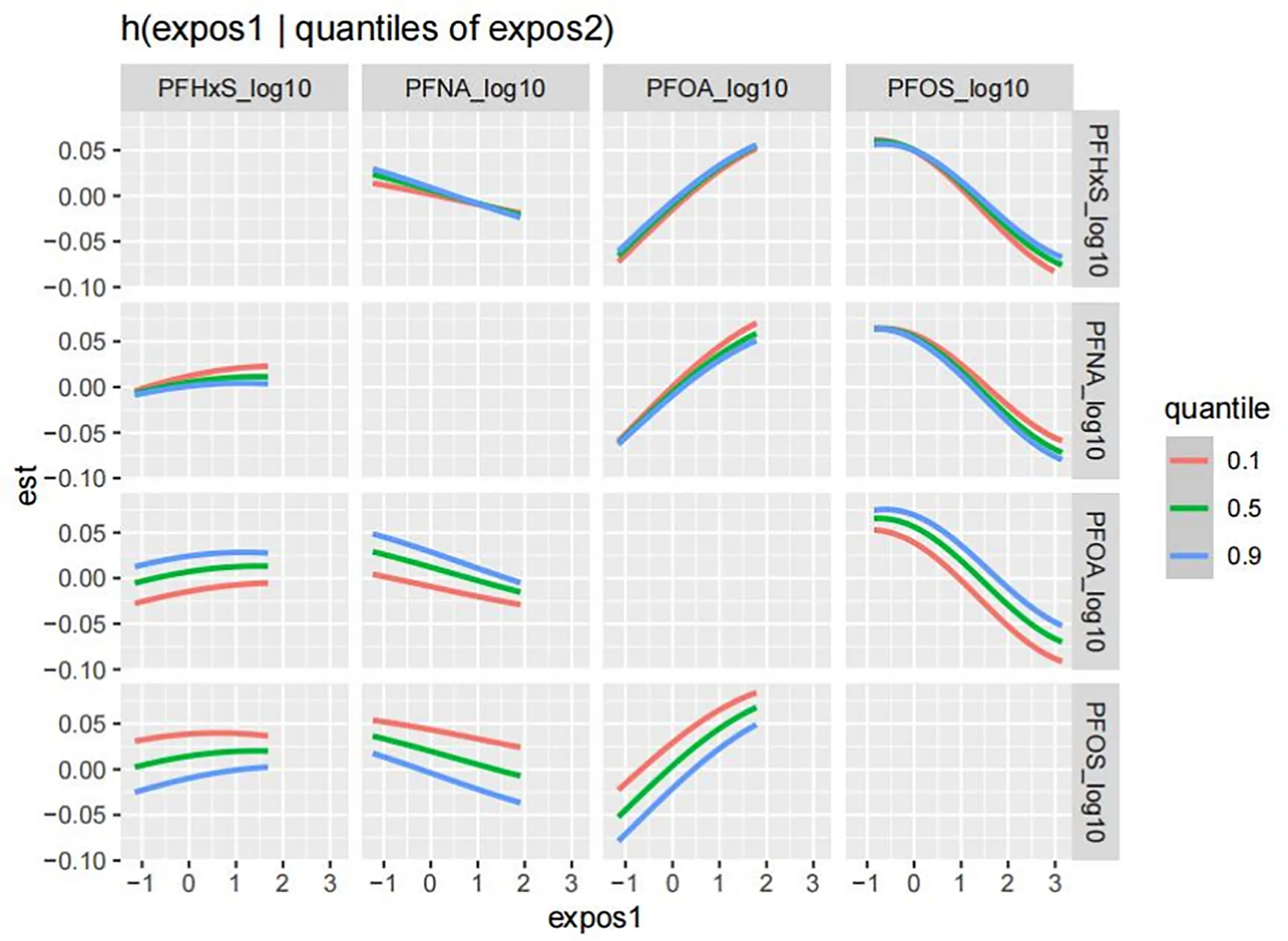
The interaction of PFAS and asthma by the BKMR model was adjusted for all covariates. BKMR = Bayesian kernel machine regression; PFAS = per- and polyfluoroalkyl substances.

The univariate exposure-response functions for the 4 PFAS, with other PFAS fixed at their 50th percentile levels, revealed a clear negative relationship between PFOS and PFNA and asthma risk, while PFOA exhibited an increasing trend (Fig. 5). Additionally, bivariate exposure-response functions were plotted for each PFAS metabolite at the 10th, 50th, and 90th percentiles, with other PFAS held constant at the 50th percentile to explore potential interactions. In cases where the slopes of the bivariate PFAS-response functions remained consistent across different quantiles of another PFAS, no potential interactions were detected. However, a convergence of 3 PFAS curves was observed as PFAS levels increased from the 10th to the 90th percentile, suggesting a possible interaction between specific PFAS pairs (e.g., PFHxS and PFNA; PFOA and PFHxS; PFOA and PFNA; PFOS and PFHxS; PFOS and PFNA) (Fig. 6).

**Figure 7.**
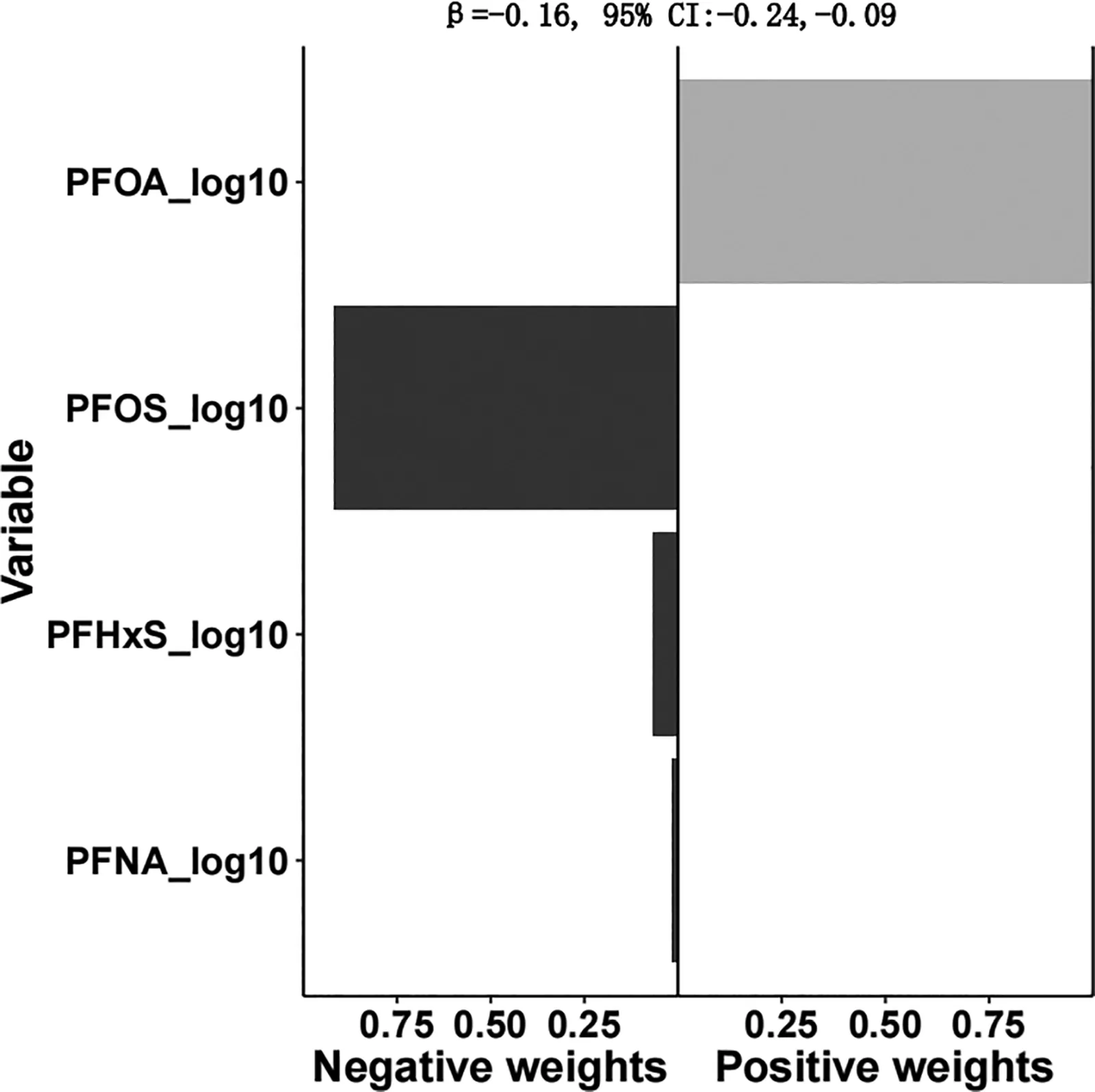
The directions and magnitude of the assigned weights for each log10-transformed PFAS in relation to asthma in quantile g-computation. PFAS = Per- and polyfluoroalkyl substances.

Q-gcomp combines the inferential simplicity of WQS with the flexibility of g-computation, allowing individual components in the mixture to contribute either positively or negatively to the overall mixture index, with the sum of all weights equaling 1. Consistent with the BKMR model results, the Q-gcomp analysis revealed a significant inverse association between the mixture of 4 PFAS and asthma (β = −0.16, 95% CI: −0.24, −0.09) (Fig. 3). Among the PFAS, PFOS contributed the most to this inverse association, accounting for 91.8% of the effect (Fig. 7).

## 4. Discussion

The prevalence and persistence of PFAS have made them ubiquitous environmental pollutants, with widespread implications for human health.^[14]^ Long-chain PFAS, in particular, can accumulate after oral exposure and reach distant organs, including the lungs.^[15]^ These organs are vulnerable to systemic and inhaled PFAS, which can alter pulmonary surfactant activity and trigger significant inflammatory responses.^[16–18]^ Our research employed 3 distinct statistical techniques to examine the impact of single or combined PFAS exposure on asthma in adult residents of the United States. These diverse statistical models provided comprehensive insights from different algorithmic perspectives. The single-pollutant logistic regression model identified a negative association between asthma and both PFOS and PFNA. Additionally, RCS analysis indicated a linear relationship between PFOS and asthma. In multi-pollutant models, the WQS index further supported a negative association with asthma, effectively addressing collinearity and nonlinearity issues inherent in logistic regression. Using BKMR, we were able to select variables from high-dimensional exposure vectors, facilitating the exploration of interactions and nonlinearity among mixture components. Both BKMR and Q-gcomp suggested an overall inverse association between the PFAS mixture and asthma, with PFOS emerging as the predominant contributor. However, these findings should be interpreted in the context of the different assumptions underlying the mixture models. Unlike WQS regression, which assumes that all mixture components contribute in the same prespecified direction, BKMR and Q-gcomp allow individual PFAS congeners to exhibit heterogeneous exposure-response relationships. Consistent with this flexibility, BKMR demonstrated that PFOS and PFNA were inversely associated with asthma, whereas PFOA showed positive associations over portions of the exposure range. Likewise, Q-gcomp indicated that PFOS accounted for 91.8% of the overall inverse mixture effect, suggesting that the observed association was driven primarily by PFOS rather than by uniformly inverse effects across all PFAS congeners. Therefore, the overall mixture effect should not be interpreted as indicating that all PFAS compounds exert similar or “protective” effects on asthma risk. Instead, these findings support heterogeneous component-specific associations, with individual PFAS congeners potentially influencing asthma through distinct biological mechanisms. These results suggest that PFOS exposure may be significantly related to the development of asthma, although further investigation into the underlying mechanisms is warranted.

Asthma is a heterogeneous disease encompassing distinct endotypes, such as type 2 (T2) and non-type 2 (non-T2) inflammation, which may differ in their underlying pathophysiology and response to environmental exposures. Numerous investigations have examined the relationship between PFAS and childhood asthma, yielding conflicting conclusions.^[19–21]^ For example, a study involving Taiwanese children aged 9 to 16 years found that exposure to PFOA and PFOS was associated with increased asthma severity,^[22]^ whereas another investigation observed no significant association between several legacy PFAS compounds and childhood asthma despite evidence of age-dependent effects.^[21]^ Similarly, prenatal exposure studies have suggested that long-chain PFAAs may exert immunosuppressive effects on allergic diseases during early childhood,^[23]^ while analyses of adolescent participants in NHANES reported inverse associations between PFOS and asthma but positive associations for PFOA.^[19]^ Collectively, these findings suggest that the relationship between PFAS exposure and asthma is complex and may vary according to exposure window, age, PFAS congener, asthma phenotype, and underlying immunological status. Compared with the relatively abundant literature in pediatric populations, epidemiological evidence regarding PFAS exposure and asthma in adults remains limited. The present study therefore extends the current evidence by characterizing both individual and mixture associations between serum PFAS concentrations and asthma in a nationally representative adult population.

Although the biological mechanisms underlying the observed inverse association remain incompletely understood, several experimental studies provide potential mechanistic explanations while simultaneously highlighting the complexity of PFAS immunotoxicity. PFOS and PFOA are among the most persistent legacy PFAS compounds, with estimated human biological half-lives exceeding 4 years, allowing prolonged accumulation following environmental exposure.^[24]^ Previous studies have demonstrated a link between PFOA, asthma, and pro-inflammatory effects.^[25–27]^ PFOS demonstrates dual effects: it activates the intrinsic immune system via AIM2 inflammasome targeting,^[17]^ induces mitochondrial oxidative stress, and promotes apoptosis in pulmonary epithelial cells,^[28]^ whereas PFOS and PFOA have also been shown to stimulate the release of pro-inflammatory cytokines, including IL-1α and IL-1β, in activated immune cells.^[16]^ In contrast, moderate levels of PFOS hinder the release of several chemokines, such as CXCL8 and CXCL10,^[16]^ and PFOS exposure is associated with decreased infiltration of OVA-induced cytokines and inflammatory cells in the lungs.^[26]^ More recently, PFOS has also been shown to directly bind to the major house dust mite allergen Der p 1, thereby reducing allergenicity and alleviating allergic airway inflammation in experimental animals.^[29]^ The varying effects on the lungs induced by these compounds likely stem from differences in their affinity for and activation of PPAR. Notably, PFOA exhibits a lower affinity for PPARγ compared to PFOS.^[30]^ PPAR-γ agonists possess anti-inflammatory properties and modulate cytokine secretion in asthma.^[31]^

Despite these experimental observations, caution is warranted when interpreting the inverse associations identified in the present study. Most mechanistic evidence has been derived from in vitro experiments or animal models employing exposure concentrations substantially higher than those encountered in the general population. Whether these mechanisms operate under environmentally relevant human exposure conditions remains uncertain, and direct mechanistic evidence in adult populations is currently lacking. Moreover, PFAS are widely recognized as persistent environmental contaminants with established immunotoxic properties, and the overall toxicological literature predominantly supports adverse rather than beneficial effects on immune regulation and respiratory health. Therefore, the inverse associations observed in this cross-sectional study should not be interpreted as evidence that PFAS exposure protects against asthma. Rather, they should be regarded as epidemiological observations that warrant confirmation through prospective cohort studies and mechanistic investigations conducted under environmentally relevant exposure conditions.

Interestingly, the RCS for 4 PFAS congeners were not consistent with dose-response curves in BKMR; the differences in results between RCS and BKMR may be due to BKMR’s ability to account for interactions and co-exposures, which RCS might not fully capture. Additionally, RCS’s reliance on parametric choices, such as the selection of knots, can significantly impact the shape of the curve, leading to differences from BKMR’s data-driven approach. These discrepancies highlight the inherent differences in the methods, but both offer valuable insights. Together, they provide a more comprehensive understanding of the data. Furthermore, although we observed a relationship between PFOS and asthma in adults, the evidence was relatively weaker compared to non-asthmatics, resulting in larger standard errors and reduced precision in effect estimates.

Several limitations should be considered when interpreting our findings. First, the cross-sectional design of NHANES precludes the establishment of temporal or causal relationships between PFAS exposure and asthma. Although inverse associations were consistently observed across multiple analytical approaches, it remains unclear whether PFAS exposure preceded asthma onset or whether asthma itself influenced serum PFAS concentrations through changes in health-related behaviors, dietary habits, healthcare utilization, disease management, or medication use. Consequently, reverse causation remains an important alternative explanation that cannot be excluded. While extensive covariate adjustment and sensitivity analyses supported the robustness of the observed associations to measured confounding, they cannot eliminate bias arising from reverse causation or unmeasured confounders. Therefore, the present findings should be interpreted as epidemiological associations rather than evidence of a causal or protective effect.

Second, we observed substantial temporal heterogeneity across NHANES survey cycles. Although estimates from earlier survey periods were less precise because of relatively small sample sizes, statistical imprecision alone is unlikely to explain the observed differences in the direction and magnitude of associations before and after major PFAS regulatory actions. These temporal differences may reflect secular changes in environmental PFAS exposure profiles, shifts in co-exposure patterns, evolving asthma diagnosis and treatment practices, demographic changes in the study population, or other unmeasured environmental and societal factors. Accordingly, the pooled estimates reported in this study should not be regarded as temporally stable but rather as hypothesis-generating observations requiring confirmation in prospective studies with repeated exposure assessments across different time periods.

Additionally, the reliance on questionnaire-based asthma assessment may have introduced misclassification bias, potentially attenuating the observed associations. Although self-reported physician-diagnosed asthma is widely used in NHANES epidemiologic studies, objective pulmonary function validation was not feasible in the present study due to the substantial proportion of missing post-bronchodilator spirometry data. Future studies incorporating standardized spirometric assessment are warranted to validate these findings.

Finally, residual confounding cannot be completely excluded. Although we adjusted for a broad range of demographic, socioeconomic, lifestyle, and clinical factors, information on several potentially important co-exposures, including volatile organic compounds and other environmental pollutants, was unavailable or incomplete within the present analytical framework. Future investigations employing comprehensive multi-pollutant exposure models may provide a more complete understanding of the independent contribution of PFAS to asthma risk. In addition, our analyses primarily evaluated legacy PFAS compounds, including PFOS, PFOA, PFNA, and PFHxS. Although NHANES measured several emerging replacement PFAS compounds during the 2017 to 2018 survey cycle, including GenX, DONA, CLPF, FHPS, and PFHA, detection frequencies were extremely low, precluding reliable evaluation of their contributions to mixture effects. Consequently, caution should be exercised when extrapolating our findings to newer-generation PFAS chemicals, whose exposure characteristics and biological effects may differ substantially from those of legacy PFAS.

## 5. Conclusions

While the correlation between adult asthma and serum PFAS exposure has been inadequately explored, our study aims to address this significant research gap. Our findings indicate that although PFOA and PFHxS did not demonstrate statistically significant associations with adult asthma, PFOS was identified as a key factor associated with asthma in adults. These results suggest that further investigation is necessary to clarify the underlying mechanisms by which PFOS influences asthma development in adults.

## Acknowledgments

We thank all participants and staff of the National Health and Nutrition Examination Survey (NHANES) for their efforts in data collection and management. We also acknowledge the valuable assistance of our colleagues in statistical consultation and manuscript revision.

## Author contributions

**Data curation:** Tiankai Shan.

**Formal analysis:** Kai Wu.

**Methodology:** Kai Wu.

**Supervision:** Jingxian Jiang.

**Writing – original draft:** Tiankai Shan.

**Writing – review & editing:** Jingxian Jiang.
















