# Supplementary material for: Correlation between perfluoroalkyl and polyfluoroalkyl substances and adult asthma in the NHANES
Source: Medicine (Baltimore). 2026 Aug 28;105(35):e50225. doi: 10.1097/MD.0000000000050225 (PMC13529185; doi:10.1097/MD.0000000000050225)
Supplement: Supplementary file 1 [file medi-105-e50225-s001.docx]

**Table S1. Distribution of phthalate metabolites (N = 7106), NHANES, USA, 2003–2018.**

| PFAS | Percentile |  |  |
| --- | --- | --- | --- |
|  | 25% | 50% | 75% |
| PFOA_log10 | 0.18 | 0.41 | 0.62 |
| PFOS_log10 | 0.68 | 0.98 | 1.25 |
| PFHxS_log10 | -0.10 | 0.18 | 0.41 |
| PFNA_log10 | -0.30 | -0.05 | 0.14 |
