# Supplementary material for: Correlation between perfluoroalkyl and polyfluoroalkyl substances and adult asthma in the NHANES
Source: Medicine (Baltimore). 2026 Aug 28;105(35):e50225. doi: 10.1097/MD.0000000000050225 (PMC13529185; doi:10.1097/MD.0000000000050225)
Supplement: Supplementary file 2 [file medi-105-e50225-s004.docx]

**Table S2. Eosinophils as a mediator in the associations between PFAS and asthma.**

|  | Mediation effect | Estimate | 95% CI  lower | 95% CI  upper | *p*-value |
| --- | --- | --- | --- | --- | --- |
| PFOA | Total effect | 0.002 | -0.023 | 0.027 | 0.871 |
|  | Mediation effect | -0.001 | -0.004 | 0.001 | 0.209 |
|  | Direct effect | 0.004 | -0.022 | 0.028 | 0.782 |
|  | Proportion mediated | -0.0694 | -29.634 | -0.262 | 0.841 |
| PFOS | Total effect | -0.023 | -0.044 | -0.003 | 0.028 |
|  | Mediation effect | -0.002 | -0.004 | 0.000 | 0.084 |
|  | Direct effect | -0.021 | -0.041 | -0.001 | 0.041 |
|  | Proportion mediated | 0.072 | -0.003 | 0.501 | 0.888 |
| PFHxS | Total effect | -0.002 | -0.023 | 0.020 | 0.867 |
|  | Mediation effect | -0.003 | -0.005 | -0.001 | 0.008 |
|  | Direct effect | 0.001 | -0.019 | 0.023 | 0.920 |
|  | Proportion mediated | 0.1593 | 0.788 | 147.542 | 0.779 |
| PFNA | Total effect | -0.016 | -0.039 | 0.008 | 0.160 |
|  | Mediation effect | -0.001 | -0.003 | 0.001 | 0.491 |
|  | Direct effect | -0.016 | -0.038 | 0.008 | 0.180 |
|  | Proportion mediated | 0.044 | -0.217 | 1.480 | 0.994 |

Model was adjusted for all covariates.
