# Supplementary material for: Correlation between perfluoroalkyl and polyfluoroalkyl substances and adult asthma in the NHANES
Source: Medicine (Baltimore). 2026 Aug 28;105(35):e50225. doi: 10.1097/MD.0000000000050225 (PMC13529185; doi:10.1097/MD.0000000000050225)
Supplement: Supplementary file 3 [file medi-105-e50225-s007.docx]

**Table S3. The estimated PFAS weights of the four PFAS**

|  | mix_name | mean_weight |
| --- | --- | --- |
| Positive | PFOS_log10 | 0.743 |
|  | PFHxS_log10 | 0.120 |
|  | PFOA_log10 | 0.075 |
|  | PFNA_log10 | 0.062 |
| Negative | PFHxS_log10 | 0.729 |
|  | PFOA_log10 | 0.177 |
|  | PFNA_log10 | 0.094 |
|  | PFOS_log10 | 0.000 |
