# Supplementary material for: Correlation between perfluoroalkyl and polyfluoroalkyl substances and adult asthma in the NHANES
Source: Medicine (Baltimore). 2026 Aug 28;105(35):e50225. doi: 10.1097/MD.0000000000050225 (PMC13529185; doi:10.1097/MD.0000000000050225)
Supplement: Supplementary file 4 [file medi-105-e50225-s008.docx]

**Table S4. Posterior inclusion and conditional probabilities of Bayesian kernel machine regression models (PIP).**

|  | PIP |
| --- | --- |
| PFOA_log10 | 0.4712 |
| PFOS_log10 | 0.6288 |
| PFHxS_log10 | 0.1876 |
| PFNA_log10 | 0.2824 |
